# Genotype-Specific Detection of Measles Virus Using Wastewater Surveillance — Wisconsin, February 2026

**DOI:** 10.15585/mmwr.mm7527a1

**Published:** 2026-07-16

**Authors:** Ian Pray, Adélaïde Roguet, H. Auguste Dutcher, Stephanie Schauer, Matthew Dereadt, Megan Rasmussen, Ryan Westergaard, Dagmara Antkiewicz, Erica Henning, Devin Everett, Martin Shafer

**Affiliations:** ^1^Career Epidemiology Field Officer Program, Office of Readiness and Response, CDC; ^2^Wisconsin Department of Health Services; ^3^Wisconsin State Laboratory of Hygiene, School of Medicine & Public Health, University of Wisconsin-Madison, Madison, Wisconsin; ^4^Epidemic Intelligence Service, CDC.

**Summary**

**What is already known about this topic?**

Wastewater (sewage) surveillance has been increasingly used to support public health surveillance for infectious diseases, including measles, in the United States.

**What is added by this report?**

In February 2026, two unrelated measles cases were reported to the Wisconsin Department of Health Services. Wastewater surveillance detected measles virus associated with one case of measles caused by the D8 genotype, before the case was reported. However, the same widely used wastewater surveillance assay for measles failed to detect the virus from the second case, which was caused by a different genotype (B3). After the assay limitation was identified by public health officials, the developers released a modified assay that improved detection of the measles B3 genotype.

**What are the implications for public health practice?**

To ensure that wastewater data are as accurate and useful as possible, wastewater assays should be standardized and revalidated against circulating viruses and supported by publicly available whole-genome sequencing data.

## Abstract

In February 2026, two travel-associated cases of measles were reported to the Wisconsin Department of Health Services (WDHS). Public health investigations for each case included wastewater (sewage) surveillance for wild-type measles, which was first implemented by the WDHS Wastewater Monitoring Program in June 2025. The virus from one case, caused by measles genotype D8, was detected by a wastewater surveillance assay before the case was identified by local health authorities, leading to public health notifications and action. Measles virus from the second case, caused by measles genotype B3, was not detected by the assay. Additional analysis of the measles wastewater assay, which is among the most widely used assays in the United States, revealed that the assay was not able to detect an internationally circulating variant of the measles B3 genotype. Two alternative assays confirmed that measles virus was present and detectable in the wastewater. The assay developers designed and released a modified assay that incorporated an additional probe that could detect the B3 variant. These cases demonstrate the potential benefit of wastewater surveillance in detecting wild-type measles virus from a single case, while also highlighting the importance of regularly monitoring wastewater assays with respect to available genomic data. These activities should be supported by up-to-date libraries of publicly available whole-genome sequencing data.

## Introduction

Wastewater surveillance has become a valuable tool for improving community-level surveillance for many pathogens, including influenza, COVID-19, respiratory syncytial virus, and monkeypox and is now increasingly used for tracking measles in U.S. communities. Recent findings demonstrate that measles virus nucleic acid can be detected in wastewater before outbreaks are recognized, thereby helping public health officials respond quickly to local cases (*1*). Several commercial and published assays, adapted for the two primary digital polymerase chain reaction (dPCR) platforms (*2**,**3*), have become available since mid-2025 to monitor wild-type measles in wastewater. However, interlaboratory proficiency testing conducted by the Wisconsin State Laboratory of Hygiene in February 2026, found that performance across four of the most commonly used measles assays varied widely, highlighting the importance of regular monitoring for assay performance (Wisconsin State Laboratory of Hygiene, unpublished data, 2026).

The Wisconsin Department of Health Services (WDHS) Wastewater Monitoring Program routinely tests influent wastewater from 44 municipal wastewater treatment plants across Wisconsin, covering approximately 50% of the state’s total population served by a participating sewershed. Surveillance for wild-type measles began in June 2025, using an established measles assay (*2*). This assay was already in use by a large commercial wastewater laboratory and numerous state public health laboratories, making it one of the most widely used measles wastewater assays in the United States during 2025–2026.

## Investigation and Findings

In February 2026, two measles cases (in patients A and B, who lived in areas served by different sewersheds) were reported to WDHS (Figure). Contact investigations found that each case was travel associated, and the cases were not epidemiologically linked. WDHS used wastewater surveillance for wild-type measles to support each investigation. This investigation was reviewed by CDC, deemed not research, and conducted consistent with applicable federal law and CDC policy.*

**FIGURE:**
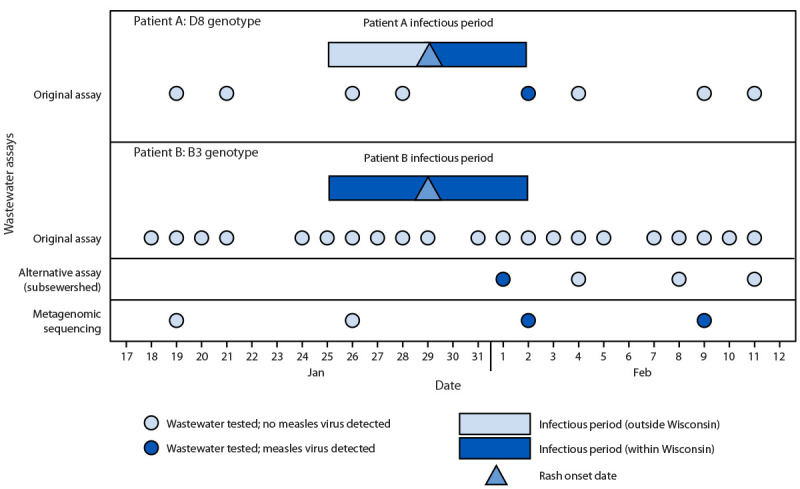
Timeline of wastewater testing results* for measles virus genotypes D8 (patient A) and B3 (patient B) using multiple assays^†^ — Wisconsin, 2026 * Dates for wastewater surveillance indicate the beginning of the 24-hour composite sample collection. ^†^ The original (standard) assay used to detect the measles virus in wastewater has been described previously (https://doi.org/10.1039/D6EW00020G). The alternative assay (https://doi.org/10.1039/d6ew00020g) is a quantitative, gene-specific, real-time reverse transcription–polymerase chain reaction assay used to detect measles virus in clinical specimens. This alternative assay, which was only used after the original assay did not detect measles virus in the wastewater samples from the wastewater treatment facility serving patient B’s residence, is not used routinely because it is not specific for wild-type measles. Alternative assay results are only shown for samples from subsewershed interceptors that collected wastewater from the area within the sewershed where patient B lived.

### Patient A

**Detection of measles virus in a wastewater sample.** On February 5, 2026, the WDHS Wastewater Monitoring Program detected wild-type measles virus in a sample collected on February 2, by a wastewater treatment plant serving approximately 30,000 residents in Wisconsin.

**Notification to WDHS of confirmed measles case.** Later the same day, WDHS was notified of a confirmed case of measles in an adult (patient A) who had returned to Wisconsin on January 29, after traveling domestically; during this period (4 days before through 4 days after rash onset), the patient was infectious. Rash onset occurred during travel (January 29), and the patient isolated at home in Wisconsin on arrival. When the positive wastewater sample was collected (February 2), patient A was still isolated at the Wisconsin residence, which was located within the sewershed where the wastewater detection occurred. Patient A’s measles case was clinically diagnosed without laboratory genotyping but was epidemiologically linked to an out-of-state measles outbreak of genotype D8, the predominant circulating measles genotype in the United States (*4*).

### Patient B

**Notification to WDHS of confirmed measles case.** On February 1, 2026, WDHS received laboratory confirmation of a measles case in a Wisconsin resident (patient B). Investigation indicated that patient B had become infected with measles while abroad, patient B’s measles case was not epidemiologically linked to patient A’s case, and patient B lived in a different Wisconsin county from patient A, which was served by a different sewershed. Sequencing of the specimen identified the virus as measles virus B3 genotype,^†^ an internationally circulating measles genotype that accounted for approximately 5%–10% of U.S. cases in 2025 (*4*). The onset of patient B’s rash occurred on January 29, and the patient remained isolated at home during the infectious period (January 25–February 2).

**Additional testing after no measles virus detected in wastewater samples.** Despite confirmation that the patient was at home during the infectious period, the standard measles wastewater assay did not detect measles virus in any of the eight wastewater samples collected during January 25–February 2, 2026, from the wastewater treatment facility serving the patient’s residence (*2*). Additional laboratory testing indicated that the measles wastewater assay was unable to detect measles virus either in the clinical isolate or in an available B3 reference strain.^§^ Follow-up testing of wastewater samples involved two independent methods. One method involved using an alternative dPCR assay for measles used by the program’s laboratory (*5*) to analyze a subsewershed sample collected on February 1, from the area where patient B lived. The second method involved using an untargeted approach known as shotgun metagenomic viral sequencing to analyze citywide wastewater samples; this approach attempts to sequence all viral genetic material in a sample and can identify novel or unexpected pathogens (*6*). Both methods confirmed the presence of measles virus at detectable levels in the wastewater samples. The metagenomic sequencing results of the wastewater sample from February 2, were identical to the whole-genome sequencing results for patient B’s clinical measles isolate,^¶^ providing strong evidence that patient B was the source of the measles virus detected in the wastewater.

## Public Health Response

### Patient A

State and local authorities were informed about the measles virus detection in wastewater, and information regarding the wastewater detection was included in a public media release. All wastewater monitoring results for measles in Wisconsin are shared with local health departments and published on CDC’s national measles wastewater dashboard. Case and contact investigations for patient A were conducted by the local health department. Known contacts were notified of potential exposure, and notices of public exposure locations were issued. No further cases were identified in connection with patient A. In addition, no measles virus was detected in wastewater samples collected before or after the initial detection, suggesting that public health measures might have effectively contained the travel-related introduction.

### Patient B

WDHS notified CDC and the assay developers that the wastewater assay was unable to detect the measles virus B3 genotype (*2*). Using publicly available whole-genome sequencing data, the assay developers identified an *N *gene mutation in recent measles virus genotype B3 samples; this mutation caused the assay failure. On March 2, the developers released a modified assay that incorporated an additional probe** to bind to the mutation and improve coverage of the assay. Case and contact investigations for patient B were conducted by the local health department. Known contacts were notified of potential exposure, and notices of public exposure locations were issued. No further cases were identified in connection with patient B.

## Discussion

These two measles cases demonstrate the benefits of and potential challenges associated with wastewater surveillance as a public health tool. The detections of measles virus in wastewater associated with patient A’s measles case adds to a growing body of evidence that wastewater surveillance can detect measles virus from a single case (in this instance, one known case in a sewershed serving approximately 30,000 persons). This high level of sensitivity underscores the potential for wastewater surveillance to detect cases or outbreaks and prompt public health action that can prevent additional cases.

The experience with the second measles case (in patient B) highlights several other considerations that are important for ensuring that wastewater surveillance continues to provide accurate and actionable data. In this case, a widely used wastewater assay for measles surveillance was unable to detect an internationally circulating variant of measles genotype (B3). The developers of the original wastewater assay (*2*) used 97 measles sequences (43 B3 sequences and 54 D8 sequences) and the measles vaccine strain (i.e., Edmonston strain), all obtained from the National Center for Biotechnology Information in March 2025, to design the assay. They performed computer (i.e., in-silico) modeling to confirm specificity to circulating measles genotypes B3 and D8. The assay design was made publicly available and has been used in many U.S. states since May 2025.

A mutation in a section of the *N *gene of the measles B3 virus, which the original assay targeted, was rarely detected before the assay was developed; <2% of available measles B3 sequences contained the mutation.^††^ However, during the period after the assay was released (May 2025–February 2026), 75% (169 of 226) of available measles B3 sequences contained the mutation. Sample collection dates for these sequences suggest that this variant has been the predominant B3 variant in international circulation since July 2024.

The CDC-validated wastewater assay for measles, adapted from a multiplexed reverse transcription-droplet dPCR assay (*3*), targets the *M* gene of the measles virus (and therefore was not affected by the *N*-gene mutation). However, jurisdictions relying on the original assay for measles surveillance might have experienced reduced sensitivity for detecting B3-associated cases or outbreaks before the assay was modified through the addition of a second probe.

This gap in surveillance for the measles B3 genotype highlights the need for improved monitoring and follow-up of wastewater assays with respect to available genomic data. Regular computer modeling and wet laboratory evaluations of assay primers and probes for circulating virus strains are critical for identifying and correcting assay probe mismatches with circulating lineages before they affect overall surveillance. These findings also highlight the importance of performance testing of wastewater assays.

With various laboratory assays available for measles wastewater surveillance, and a lack of standardized performance testing across assays, future genetic evolution of the measles virus might limit detection by current wastewater assays, affecting surveillance and requiring frequent revalidation. Ongoing assay development and updates should be supported by the availability of a robust and up-to-date library of publicly available whole-genome sequencing data. Development of sequencing-based approaches for detection of measles and other emerging public health threats could address this problem by monitoring mutating viruses without requiring updates to targeted approaches.

## Footnotes

* 45 C.F.R. part 46.102(l)(2), 21 C.F.R. part 56; 42 U.S.C. Sect. 241(d); 5 U.S.C. Sect. 552a; 44 U.S.C.

† Specimen genotyping of the sample from patient B was performed by the Wisconsin State Laboratory of Hygiene, Communicable Disease Division, using Sanger sequencing of the measles *N450* gene region.

§ Measles genotype B3 reference strain was cultured by CDC from a 2024 case sample and distributed by BEI (MVs/Tennessee. USA/40.24).

¶ Metagenomic sequencing of the wastewater sample from February 2, 2026, had five reads ranging from 92 to 148 base pairs that perfectly matched the consensus genomes from patient B in the *H* and *L* gene regions. CDC performed whole-genome sequencing of the patient sample to establish a match with the wastewater in May 2026 after conclusion of the investigation. Specimen identifiers for whole-genome sequencing, patient B: MVs/Wisconsin.USA/Jan.26/2166 and MVs/Wisconsin.USA/Jan.26/2801.

** The original assay probe was CATGATGATCC**A**AGTAGTAGTGA. For genotype B3, an adenine-to-guanine (A-to-G) mutation in the probe-binding region (nucleoprotein gene) caused the mismatch. A modified probe was added in March 2026: CATGATGATCC**G**AGTAGTAGTGA.

†† Data on measles B3 sequences were obtained from Measles Virus | Pathoplexus and the National Center for Biotechnology Information. Two samples (collected in 2020 and 2024) were identified with sequences containing the *N*-gene mutation before the original wastewater assay for measles was developed.
